# Patent Foramen Ovale in Children: A Review of Epidemiological Characteristics, Natural Course, and Intervention Strategies

**DOI:** 10.3390/children12111491

**Published:** 2025-11-04

**Authors:** Congcong Wang, Lianglong Ma, Jie Jin, Zhuo Shi

**Affiliations:** Department of Cardiac Surgery, The Children’s Hospital, Zhejiang University School of Medicine, National Clinical Research Center for Child Health, Hangzhou 310052, China; 22318601@zju.edu.cn (C.W.); malianglong@zju.edu.cn (L.M.); jinjie0910@zju.edu.cn (J.J.)

**Keywords:** patent foramen ovale, children, epidemiology, natural course, indications, timing of intervention, complications

## Abstract

**Highlights:**

**What are the main findings?**

- The incidence of patent foramen ovale in children is relatively high, particularly in preterm infants and children with specific genetic syndromes.
- Delayed intervention is recommended to avoid unnecessary procedural risks for asymptomatic children with patent foramen ovale.

**What is the implication of the main finding?**

- The high incidence yet benign nature of patent foramen ovale requires increased awareness and screening to identify high-risk cases needing intervention.
- Intervention strategies require individualized risk stratification, favoring monitoring for most but intervening for complications like stroke.

**Abstract:**

Patent foramen ovale (PFO) is a common cardiac structural abnormality in children, which has garnered significant clinical attention due to its epidemiological characteristics and natural course. Although PFO is asymptomatic in many cases, its potential complications and impact on children’s health should not be underestimated. In recent years, there has been an increase in research on the epidemiological data of PFO in children under 16 years old. However, controversy remains regarding its natural course and clinical intervention strategies. This paper reviews the latest research progress on PFO in terms of epidemiological characteristics, natural course, current interventions, indications, timing of percutaneous PFO closure, risks, and complications, aiming to provide evidence-based guidance for clinical decision-making and improved management strategies for children with patent foramen ovale.

## 1. Introduction

Patent foramen ovale (PFO) is a normal physiological channel during fetal life that usually closes within the first year after birth, but in some children, the foramen ovale fails to close naturally, resulting in PFO. This condition is relatively common in children, and studies have shown that PFO may be associated with various clinical symptoms, including cryptogenic stroke and migraine [1,2]. Therefore, the epidemiological characteristics, natural course, and intervention strategies related to PFO have gradually become a focus of clinical research. With the advancement of imaging technology and interventional treatments, significant progress has been made in the diagnosis and treatment of PFO.

Although stroke is rare in the pediatric population, studies have shown that PFO is more prevalent in children with cryptogenic stroke than in normal children [3,4]. According to one study, in children undergoing stroke evaluation, the incidence of PFO among cryptogenic stroke patients was 28%, significantly higher than that of patients with other known causes of stroke and healthy controls [5]. Additionally, PFO may also be related to other clinical syndromes, such as migraine and potential cardiac complications. This association further raises concerns about PFO in children [2,6].

Regarding the natural course, most healthy children with PFO typically do not require intervention, as many cases show that PFO does not lead to serious health issues during follow-up [1]. However, in certain cases, particularly those associated with cryptogenic stroke, consideration may need to be given to performing percutaneous PFO closure. Multiple studies have indicated that percutaneous PFO closure in specific children can significantly improve symptoms, especially after experiencing migraines or strokes [7,8].

In terms of intervention strategies, recent studies have provided new insights into the management of PFO. While closure strategies for PFO in adults are relatively mature, the best management approach for children remains unclear. In patients with high-risk clinical syndromes, such as sickle cell disease and other cardiac conditions, the presence of PFO may increase the risk of stroke, making assessment and management of PFO particularly important [9]. Furthermore, imaging examinations play a crucial role in the diagnosis of PFO in children, with echocardiography being considered the most appropriate examination method due to its ease of operation, lack of need for anesthesia, and suitability for the unique needs of children [2].

## 2. Epidemiological Characteristics of Pediatric PFO

### 2.1. Incidence and Influencing Factors

Globally, the incidence of patent foramen ovale (PFO) in children is approximately 25–30%. Although these data are widely accepted, specific incidence rates show significant variation across different regions and populations. For example, a study in China indicated that PFO is more common among patients with congenital heart disease (CHD), particularly in low birth weight (LBW) and very low birth weight (VLBW) children, closely related to the overall health status of these children and other potential cardiac lesions [10]. Premature infants have a higher incidence of PFO due to the immaturity of physiological processes that facilitate closure, such as pulmonary development and the associated changes in cardiopulmonary hemodynamics at birth. Research indicates that the incidence of PFO in premature infants can be as high as 60–80% [11]. Furthermore, certain genetic syndromes, such as Down syndrome, are also associated with an increased incidence of PFO. These syndromes often accompany various congenital heart diseases, making PFO a common cardiac structural abnormality, particularly prevalent among these patients [12].

Regarding gender differences, current studies indicate that there is no significant gender bias in the occurrence of PFO. Although some studies suggest that the proportion of delayed closure of PFO may be slightly higher in male children, overall gender differences are not pronounced [13]. This finding suggests that clinicians should focus more on the specific health conditions and birth backgrounds of patients when assessing pediatric PFO, rather than relying solely on gender for risk assessment.

In summary, the incidence of pediatric PFO is influenced by various factors, including birth weight, mode of delivery, genetic factors, and gender. Further research and epidemiological studies on PFO will help better understand its pathogenesis and influencing factors, providing a more solid basis for clinical management.

### 2.2. Diagnostic Methods and Detection Rates

Transthoracic echocardiography (TTE) is widely regarded as the preferred tool for diagnosing patent foramen ovale (PFO) in children. This non-invasive method is highly operable and provides real-time imaging, which enables effective assessment of cardiac structure and function. However, the detection rate of TTE largely depends on the experience and skill of the operator, especially in pediatric patients. This is because the cardiac structures of children are relatively small, and due to physiological and pathological changes, the anatomical features of the heart differ from those of adults, complicating the imaging assessment. Studies have shown that the accuracy of novice operators in identifying PFO is significantly lower compared to skilled operators, which may lead to missed diagnoses or misdiagnosed cases, affecting clinical decision-making and subsequent management [2].

Contrast-enhanced ultrasound (bubble study) is an effective diagnostic method that significantly improves the detection rate of PFO, particularly in cases with small shunts. This technique involves the intravenous injection of a bubble contrast agent and requires the patient to perform actions such as coughing or holding their breath. If bubbles can pass through the PFO into the left atrium, it provides evidence of right-to-left shunting in the ultrasound imaging. This method is gradually being widely adopted in clinical practice because, compared to traditional TTE, it can more accurately identify small right-to-left shunts, particularly in children who exhibit obvious symptoms but have normal TTE results [1].

Although transesophageal echocardiography (TEE) is widely used for PFO evaluation in adults, its application in children is limited and typically reserved for complex cases or preoperative assessments. TEE provides higher-resolution images, allowing for clearer visualization of the anatomy of the atria and valves, especially in the presence of structural heart disease or other complex conditions. However, as this technique requires anesthesia and has a higher operational difficulty, careful consideration is needed when implementing it in children. Generally, TEE is mainly used for patients whose TTE results are unclear or for preoperative assessment for percutaneous PFO closure. Research indicates that while TEE can provide more information, its use in children should emphasize individualized assessment to balance risks and benefits [7].

## 3. Natural Course of Pediatric PFO

### 3.1. Natural Closure Rates and Time Frame

Research on the natural closure rates and time frame of patent foramen ovale (PFO) among pediatric populations provides important guidance for clinical intervention. Most PFOs close spontaneously within 6 to 12 months after birth, a phenomenon that has been widely confirmed in clinical observations. As age increases, particularly after the age of one, the closure rate of PFO significantly decreases. Studies have shown that the natural closure rate of PFO in children over the age of five is less than 10% [6].

Further analysis indicates that the mechanism of natural closure may be related to the development of cardiac structures and changes in hemodynamics. Significant changes in cardiac compliance and blood flow patterns occur during the first year after birth, facilitating the closure of PFO during this stage. Additionally, some studies suggest that PFO is related to certain symptoms in children (such as migraines and ischemic strokes), indicating that these potential risk factors need to be considered when evaluating the clinical significance of PFO [8,14].

While spontaneous closure is common in infancy, intervention is warranted in children with a confirmed PFO who experience a cryptogenic ischemic stroke. In these cases, percutaneous closure is recommended over medical management alone to reduce the risk of recurrence. Therefore, timely assessment and necessary interventions are extremely important for these patients. Existing cases indicate that implementing PFO closure in children related to ischemic stroke can effectively reduce the risk of subsequent strokes [15].

Thus, research related to the natural closure rate of PFOs and the time window for closure not only provides baseline data for understanding the physiological characteristics of PFO but also offers a basis for clinical decision-making, aiding doctors in making more reasonable treatment choices in pediatric cardiology management.

### 3.2. Factors Affecting Natural Outcomes

In the natural course of PFO in children, multiple factors significantly influence its closure rate and time.

Firstly, the size of the shunt is an important factor affecting closure. Studies have shown that small shunts (less than 2 mm in diameter) have a significantly higher closure rate than large shunts (greater than 4 mm in diameter). This may be related to the smaller hemodynamic impact of small shunts, leading to lower right heart load and fewer adaptive changes in cardiac structure. Additionally, large shunts may cause significant right ventricular dilation and dysfunction, thus delaying the possibility of natural closure [16].

Secondly, associated cardiac malformations are considered important factors influencing the natural closure of PFO. For example, atrial septal defect (ASD) is a common structural heart defect that may lead to hemodynamic changes, thereby hindering the closure process of PFO. Studies have shown that patients with associated ASD typically require a longer time to achieve natural closure of PFO and may even need intervention to address the associated structural issues [17]. Therefore, when evaluating the natural course of PFO in children, it is essential to consider the presence of other cardiac malformations to develop individualized intervention strategies.

Finally, genetic factors contribute significantly to the persistence and natural history of PFO. Epidemiological evidence indicates a heritable component, with a family history of PFO being an independent predictor for the presence of a right-to-left shunt [18]. At a molecular level, mutations in specific genes involved in cardiac septation can disrupt normal atrial development. For instance, mutations in the NKX2-5 gene, a critical cardiac transcription factor, have been identified in families with atrial septal defects (ASDs) and are also implicated in the pathological spectrum that includes PFO. Research demonstrates that NKX2-5 mutations can lead to a failure of the septum primum and secundum to adhere properly, thereby influencing whether the foramen ovale closes after birth or remains patent [19]. Identifying this history should lower the threshold for echocardiographic monitoring beyond infancy and prompt consideration of genetic counseling in syndromic cases.

In summary, the factors influencing the natural course of PFO in children are complex and diverse, including shunt size, associated cardiac malformations, and genetic factors. These factors interact and jointly determine the natural closure rate and time of PFO.

## 4. Current Interventions for Pediatric PFO

### 4.1. Pharmacological Interventions

The use of pharmacological interventions in the management of pediatric PFO is somewhat controversial, particularly regarding the application of antiplatelet drugs (such as aspirin) in asymptomatic children with PFO. Although some studies suggest that aspirin may have potential benefits in reducing thrombus formation and related complications, there is currently insufficient evidence to confirm its exact effects in asymptomatic children with PFO. Studies have indicated that many asymptomatic PFO patients do not require pharmacological intervention, as in most cases their natural course is favorable, and long-term use of medication may pose unnecessary risks and side effects [20]. Furthermore, the efficacy assessment of antiplatelet drugs remains inadequate, and clinicians should carefully consider the specific circumstances of patients when formulating treatment plans.

Additionally, anticoagulant therapy in children with PFO is primarily limited to those at high risk for thromboembolic events. For these patients, anticoagulant therapy can effectively reduce the risk of recurrent thrombosis. Relevant literature reports that in specific situations, such as post-cardiac surgery or children with a history of thrombosis, anticoagulant therapy can significantly improve clinical outcomes. However, the implementation of this treatment plan must be conducted under the guidance of specialized physicians to ensure appropriate drug selection and monitoring, avoiding potential bleeding risks and drug interactions [21]. Therefore, while anticoagulant therapy is feasible for some high-risk PFO patients, it is not necessary for most asymptomatic PFO children, and further research is needed to clarify its long-term safety and efficacy.

### 4.2. Interventional Treatments

Percutaneous closure is the primary intervention for children with PFO. This technique is widely recognized for its minimally invasive nature and quick recovery. Recent clinical studies have shown that this interventional treatment demonstrates significant efficacy in children with severe symptoms, especially in cases related to migraines or cryptogenic strokes. For example, a study indicated that among 46 children undergoing PFO closure, approximately 80% experienced complete relief from migraine symptoms within 12 months after closure, providing strong support for the interventional treatment of PFO [7,8]. Additionally, the incidence of complications from the closure procedure is relatively low, with many cases showing no significant residual shunt or other complications, further enhancing its application prospects in pediatric patients.

Using a catheter for PFO closure, the device is directly delivered to the heart and positioned at the foramen ovale for closure. As this process does not require open-heart surgery and is considered minimally invasive, the postoperative recovery time for patients is significantly shortened, typically allowing discharge within two days post-closure [2]. Compared to traditional surgical methods, interventional treatment not only reduces the risk of postoperative infections but also minimizes physical trauma to patients, making it particularly suitable for children with poor baseline health conditions.

Despite the widespread clinical application of percutaneous closure, it should be noted that not all PFO patients require interventional treatment. For most asymptomatic children, PFO is typically regarded as a physiological variation that does not necessitate intervention [22].

In recent years, with continuous advancements in medical technology, the development of new closure devices has brought new hope for the interventional treatment of PFO. The introduction of bioabsorbable closure devices marks a significant breakthrough in this field. The advantage of these new devices lies in their ability to effectively close PFO while gradually being absorbed by the body, thereby reducing the risk of complications associated with long-term implants, such as infections and thrombus formation [1,2]. Preliminary studies indicate that these bioabsorbable closure devices exhibit good safety and efficacy in pediatric patients.

Despite the promising clinical application prospects of bioabsorbable closure devices, their long-term efficacy still requires further research for validation. Existing clinical data are mostly short-term observations, lacking large-scale, long-term follow-up studies. To ensure the reliability of this new closure device in practical applications, future research should focus on collecting and analyzing long-term follow-up data to assess its applicability and effectiveness in children of different age groups [23].

With the development of technology and the introduction of new closure devices, interventional treatment for pediatric PFO is moving towards a safer and more effective direction. However, clinicians must still carefully evaluate each patient’s specific circumstances when implementing this treatment, integrating the latest research findings to optimize treatment plans and ensure patient safety and health.

## 5. Interventional Treatment Indications for Pediatric PFO

PFO is a common structural cardiac abnormality. Currently, treatment guidelines for adult PFO are relatively clear, but intervention guidelines for pediatric foramen ovale have yet to be established.

### 5.1. Paradoxical Embolism Leading to Cerebral Infarction

In most cases, pediatric PFO is asymptomatic, but in certain specific circumstances, it may be associated with severe clinical events, such as paradoxical embolism. Paradoxical embolism refers to the right-to-left shunt through a patent foramen ovale, leading to thrombus, gas bubbles, or other particulate matter entering the arterial system from the venous system, subsequently causing complications such as stroke or peripheral embolism. Relevant studies indicate that PFO is closely related to cryptogenic stroke, especially in children, where approximately 30% of pediatric strokes are considered cryptogenic, meaning no clear cause is identified [24]. Therefore, for children with PFO accompanied by paradoxical embolism (cryptogenic stroke) and excluding other causes, performing percutaneous closure is an absolute indication.

For these children, percutaneous PFO closure can significantly reduce the risk of stroke recurrence. Research has shown that children who underwent PFO closure did not experience new neurological symptoms during follow-up, and their quality of life improved [7]. Additionally, the success rate of percutaneous PFO closure is very high, and it is generally considered safe among children, with a lower incidence of postoperative complications compared to adults [25]. Therefore, timely identification and consideration of PFO closure are crucial for pediatric patients with stroke or peripheral embolism.

### 5.2. Special Clinical Syndromes

Platypnea–orthodeoxia syndrome is a rare clinical condition characterized by low oxygen saturation when the patient is upright or sitting, while symptoms alleviate in a supine position. This phenomenon is often associated with PFO, especially when right-to-left shunting leads to decreased arterial blood oxygen saturation. In children, Platypnea–orthodeoxia syndrome may worsen due to PFO, resulting in severe hypoxemia that affects their daily life and development. Literature reports indicate that children with PFO may exhibit significant hypoxemic symptoms after activity, with this situation being more severe in an upright position [1]. For children with PFO accompanied by severe hypoxemia, early interventional treatment is particularly important and should be considered [1].

Multiple studies have confirmed that after PFO closure, patients show significant improvements in oxygenation levels, and no new hypoxemia events were observed during the postoperative follow-up period [22]. Therefore, for children with PFO accompanied by platypnea–orthodeoxia syndrome, especially those who have not achieved significant improvement under conservative treatment, PFO closure is considered an absolute indication.

Decompression sickness is a common complication after diving activities, and the presence of PFO significantly increases the risk of gas embolism, which can lead to serious health issues [26]. During diving, pressure changes can lead to the formation of bubbles within the body, which may trigger decompression sickness. Symptoms of decompression sickness include joint pain, rash, and neurological damage, and in some cases, symptoms may persist even after hyperbaric oxygen therapy [27]. Additionally, divers with PFO are at greater risk of gas embolism during decompression, leading to dive-related complications that affect their quality of life [26]. Therefore, timely identification and management of decompression sickness are crucial, especially for patients with PFO after diving.

Patients with sickle cell disease and PFO face a higher risk of paradoxical embolism, which is particularly significant. Due to the unique hematopathological state of sickle cell disease, patients often have a higher risk of thrombosis, and this risk is further increased when accompanied by PFO. Studies have shown that the incidence of PFO in healthy populations is about 25%, but in patients with sickle cell disease, due to altered blood flow and a tendency for thrombosis, the frequency of paradoxical embolism events may be higher. This situation not only increases the risk of stroke but may also lead to other serious complications, such as myocardial infarction or peripheral vascular embolism [28,29]. Therefore, for patients with sickle cell disease, particularly those who have experienced thrombotic events, PFO screening and assessment are particularly important to allow for timely intervention to reduce the risk of paradoxical embolism [30].

### 5.3. Severe Migraine (When Other Treatments Are Ineffective)

In clinical practice, patent foramen ovale (PFO) is often regarded as an asymptomatic congenital heart defect, with many children being found to have PFO during routine echocardiography. However, when the shunt flow of PFO exceeds 4 mm and is accompanied by increased right heart load, this situation requires heightened attention. This is because larger shunt flows may lead to increased pressure load on the right ventricle, further affecting cardiac function and the overall health of children. Research indicates that PFO is associated with various clinical complications, including but not limited to migraines, cryptogenic strokes, and other cardiovascular events [13]. Therefore, for asymptomatic children with increased right heart load, further evaluation and treatment are recommended.

Research shows that percutaneous PFO closure can significantly reduce the incidence of complications associated with increased right heart load, especially in cases of right-to-left shunt. Studies have indicated that closure of PFO can effectively reduce the occurrence of recurrent cerebral ischemic events, particularly in pediatric populations [15]. Therefore, for those asymptomatic children with larger shunt flows and increased right heart load, PFO closure may be a reasonable treatment option.

Migraine is a common neurological disorder, and recent studies have found a close association between patent foramen ovale (PFO) and specific types of migraine, especially migraines with aura. For those with a history of PFO-related migraines and ineffective drug treatment, PFO closure can be considered as a treatment option. Research indicates that among patients undergoing PFO closure, the frequency and intensity of migraines significantly decrease [31].

In a retrospective study assessing 35 children with migraines and associated PFO, results showed that 80% of patients achieved complete relief at 12 months follow-up after percutaneous PFO closure, and most patients also saw a significant reduction in attack frequency [8]. These results suggest that for those children with ineffective drug treatment, PFO closure not only alleviates migraine symptoms but may also improve quality of life. Therefore, percutaneous PFO closure is a consideration for children with a history of PFO-related migraines and ineffective drug treatment.

### 5.4. Cautions

According to adult patent foramen ovale intervention guidelines, patients with simple patent foramen ovale who have not developed complications typically do not require intervention [32]. This is because, in such cases, the risk of long-term complications is extremely low, a viewpoint supported by multiple clinical studies indicating that, without other risk factors, a simple foramen ovale may not significantly affect patient health [33,34]. Therefore, for these patients, a strategy of observation and regular follow-up is often a more reasonable choice.

There is no clear association between PFO and unexplained syncope, non-aura migraines, chest pain, or palpitations; thus, interventional treatment is not recommended [15].

## 6. Timing of Interventional Treatment for Pediatric PFO

### 6.1. Pros and Cons of Early Intervention

Early intervention has been a topic of great interest in the management of pediatric patent foramen ovale (PFO). When considering the timing of intervention, it is necessary to weigh the benefits of intervention against the possibility of natural closure. Research shows that most children can close their PFO naturally during the preschool years, especially those who are asymptomatic. For these children, premature intervention may lead to unnecessary medical procedures and psychological burden [22].

### 6.2. Necessity of Early Intervention

For high-risk children meeting the aforementioned indications, the necessity for intervention becomes more urgent. Among these high-risk children, particularly those who have experienced thromboembolic events, early intervention is recommended to lower the potential risks of disability and mortality [35]. After acute cerebral infarction, anticoagulation therapy should be administered for 3–6 months before reevaluating the necessity of PFO closure to reduce the risk of recurrent stroke [36].

### 6.3. Delayed Intervention Strategies and Elective Intervention

For asymptomatic children, especially those found to have PFO after birth, clinical recommendations typically suggest delaying intervention to observe their natural course. By regularly monitoring these children’s cardiac conditions, heart structure changes can be effectively assessed, ensuring timely intervention when necessary.

Generally, for children undergoing elective percutaneous PFO closure, it is recommended that they be ≥10 years old and of suitable weight (typically over 25 kg) to reduce the risk of vascular complications [37]. In a study conducted in France, the median age for patients receiving this procedure was reported to be 14.9 years [38]. If patients have other structural heart anomalies (e.g., atrial septal aneurysm), individualized assessment is necessary to ensure optimal treatment outcomes [33].

In clinical practice, doctors need to communicate thoroughly with parents, explaining the potential benefits and drawbacks of intervention and the strategy of delayed intervention, to help them understand the importance of observation and follow-up, as well as under what circumstances intervention is needed. Parental cooperation and understanding are crucial for the health management of children, especially in cases requiring long-term observation.

## 7. Risks and Complications of Percutaneous PFO Closure

### 7.1. Intraoperative Risks

In the interventional treatment of pediatric patent foramen ovale, intraoperative risks are a significant aspect that cannot be overlooked, especially in younger children, where the incidence of vascular injury is higher. For example, femoral vein rupture is one of the common intraoperative complications, which may lead to severe bleeding and subsequent complications in younger patients. According to literature reports, despite advancements in cardiac surgical techniques for children, the risk of vascular injury remains high in younger patients undergoing interventional treatment due to anatomical structural differences and the operator’s experience level [39]. This aligns with the previously mentioned strategy of delayed intervention. During the implantation of a circular hole occluder, detachment or displacement of the occluder is a common intraoperative risk. This situation typically arises in cases with significant shunting or complex anatomical structures of the patient. The selection of the occluder and its implantation technique are crucial for the success of the procedure. Failure to effectively assess the patient’s anatomical features may lead to the occluder not being securely fixed in the target position, resulting in detachment or displacement issues. This not only affects the effectiveness of the procedure but may also increase the difficulty and risk of subsequent interventions [40].

Therefore, preoperative assessment and intraoperative monitoring are particularly important. The surgeon should have a thorough understanding of the patient’s medical history and relevant cardiac structures to ensure that timely measures can be taken during the intervention to reduce intraoperative risks. Postoperative observation is equally important, and detailed follow-up of the patient should be conducted to monitor potential risks and complications that may arise.

### 7.2. Postoperative Complications

In the interventional treatment of pediatric patent foramen ovale, the incidence of postoperative complications and their influencing factors have received extensive attention. Studies show that arrhythmias, particularly atrioventricular conduction block, have a postoperative incidence of about 1–3%, most of which are temporary [38]. These arrhythmias usually do not require special intervention, and the heart rhythm can gradually return to normal during the postoperative recovery. Therefore, continuous follow-up and monitoring are very necessary.

Moreover, thrombosis and embolic events are relatively rare after pediatric PFO occlusion, closely related to the type of occluder used and the postoperative anticoagulation strategy [41]. In some studies, patients who used anticoagulants such as aspirin or other anticoagulants showed a significantly lower risk of thrombosis after closure [42]. For instance, in a study involving children undergoing PFO occlusion, 80.5% of patients received aspirin treatment postoperatively, and 12.2% were on anticoagulants, with no significant thrombotic events or embolic complications occurring [38].

It should be noted that while the incidence of immediate postoperative complications is low, the concern for long-term complications should not be overlooked. Issues like occluder wear and endocarditis may arise years after PFO closure, necessitating long-term follow-up and monitoring of patients. Relevant studies indicate that the long-term outcomes of occluders are generally good, but attention should still be paid to their biocompatibility and potential inflammatory responses [39]. Therefore, doctors should particularly monitor changes in cardiac function and the status of the occluder during postoperative follow-up to detect potential complications early [43].

In summary, the management of postoperative complications involves not only short-term cardiac monitoring and the choice of anticoagulation strategies but also long-term follow-up and monitoring of occluder-related complications.

## 8. Discussion

In the management of pediatric patent foramen ovale (PFO), although relevant treatment guidelines have yet to be established, research on the epidemiological characteristics, natural evolution, and intervention strategies of pediatric PFO provides important evidence for clinical decision-making.

For most children, PFO often closes naturally; however, for high-risk patients, especially those who have experienced thromboembolic events or have significant shunting, timely intervention becomes particularly important. Through a comprehensive analysis of existing clinical studies, while most PFO cases do not require intervention, appropriate intervention measures in specific situations can significantly improve patient outcomes.

Transcatheter occlusion has emerged as a safe and effective treatment option, with increasing applications in pediatric PFO treatment in recent years. However, individualized assessment of the timing and indications for percutaneous PFO closure is crucial. Each patient’s specific situation varies, and physicians must consider the patient’s age, symptoms, risk of complications, and potential long-term consequences when deciding whether to intervene. This individualized assessment not only enhances treatment efficacy but also minimizes risks, which is especially important in pediatric patients.

This review is inevitably constrained by the inherent limitations present within the available literature on pediatric PFO. The primary challenge stems from the scarcity and heterogeneity of high-quality, robust data. A significant portion of the evidence synthesized herein is derived from small-scale, single-center observational studies and case series, which limits the generalizability of the findings and the strength of clinical recommendations. While this review strives to synthesize the best available evidence to guide clinical practice, these fundamental limitations in the extant literature must be acknowledged when interpreting its conclusions.

Future research directions on patent foramen ovale should focus more on evidence-based research for intervention strategies in asymptomatic children, long-term efficacy of new occluders, and genetic mechanisms of pediatric PFO. Current studies lack multi-center, large sample-sized evidence to support decision-making regarding intervention indications and timing for asymptomatic PFO patients. While existing occlusion techniques are effective, further optimization is required to enhance their safety and efficacy. Understanding the genetic basis of PFO can provide theoretical support for screening high-risk children, allowing for preventive measures in the early stages to avoid potential severe complications. Furthermore, with advancements in biotechnology, the emergence of new occluders may offer more options for patients, and the clinical trial results of these new technologies will provide important data support for future intervention strategies.

In synthesizing various studies on pediatric patent foramen ovale, it should be recognized that the management of PFO is not merely a technical issue but a comprehensive clinical decision-making process. Discrepancies and even contradictions between different study results may exist due to various factors, including sample size, study design, and patient selection. Thus, clinicians should adopt critical thinking when referencing literature, integrating their clinical experience and the specific circumstances of their patients to make decisions that best serve patients’ interests.

## 9. Conclusions

The management of pediatric PFO requires ongoing multidisciplinary collaboration and research investment, seeking evidence-based support to avoid expanding intervention indications, enhance clinical treatment outcomes, and reduce complications. Only through the integration of deeper scientific research and clinical practice can personalized and effective treatment plans be provided for every child. Future research should not only focus on technological advancements but also emphasize the overall health of patients, ensuring that every decision positively impacts children’s quality of life and health.

## Data Availability

Not applicable.

## Abbreviations

PFO	Patent Foramen Ovale
CHD	Congenital Heart Disease
LBW	Low Birth Weight
VLBW	Very Low Birth Weight
TTE	Transthoracic Echocardiography
TEE	Transesophageal Echocardiography
ASD	Atrial Septal Defect
